# Microbial response to acid stress: mechanisms and applications

**DOI:** 10.1007/s00253-019-10226-1

**Published:** 2019-11-26

**Authors:** Ningzi Guan, Long Liu

**Affiliations:** 1 Synthetic Biology and Biomedical Engineering Laboratory, Biomedical Synthetic Biology Research Center, Shanghai Key Laboratory of Regulatory Biology, Institute of Biomedical Sciences and School of Life Sciences, East China Normal University, Dongchuan Road 500, Shanghai, 200241 China; 2 Key Laboratory of Carbohydrate Chemistry and Biotechnology, Ministry of Education, Jiangnan University, Wuxi, 214122 China

**Keywords:** Acid stress, Resistance mechanism, Probiotics, Organic acids, Systems and synthetic biology

## Abstract

Microorganisms encounter acid stress during multiple bioprocesses. Microbial species have therefore developed a variety of resistance mechanisms. The damage caused by acidic environments is mitigated through the maintenance of pH homeostasis, cell membrane integrity and fluidity, metabolic regulation, and macromolecule repair. The acid tolerance mechanisms can be used to protect probiotics against gastric acids during the process of food intake, and can enhance the biosynthesis of organic acids. The combination of systems and synthetic biology technologies offers new and wide prospects for the industrial applications of microbial acid tolerance mechanisms. In this review, we summarize acid stress response mechanisms of microbial cells, illustrate the application of microbial acid tolerance in industry, and prospect the introduction of systems and synthetic biology to further explore the acid tolerance mechanisms and construct a microbial cell factory for valuable chemicals.

## Introduction

In the process of evolution, microorganisms have optimized growth conditions for their cellular functions. Metabolic disorders, and even cell death, can be caused by changes in the external environment such as pH (Beales 2004). Most microorganisms are able to survive and adapt to minor changes in environmental pH, while induced acid tolerance may occur as the environmental pH declines gradually. The role of microorganisms in human life is two-sided. Some microorganisms are pathogenic and undesirable because they develop tolerance to acid stresses by adopting preventive measures (Mani-Lopez et al. 2012). In other microorganisms, such as those used in probiotics, better acid tolerance mechanisms are desired for better physiological functions (Ranadheera et al. 2014). As an environmentally friendly and renewable process, microbial synthesis of many valuable products through fermentation has become an ideal substitution for traditional synthetic methods such as chemical and enzymatic synthesis. Higher acid tolerance adopted by microbial producers enhances their stability during the synthetic process in which acids accumulate (Hasunuma et al. 2011; Lipscomb et al. 2012). In both cases, namely, undesired pathogenic organisms and desired microbial producers, understanding of the underlying mechanisms of acid tolerance is vital for further applications of these microorganisms.

Organic acids are formed during most microbial fermentation processes as either products or by-products. The environment for microbial growth is acidified with the accumulation of organic acids, usually negatively affecting the productivity and titer of bioprocesses as the acids reach increasing concentrations (Ghaffar et al. 2014; Jiang et al. 2015; Wang and Yang 2013; Yáñez et al. 2008). The protonated acids may enter the cells and then dissociate into proton and corresponding ion, which leads to the increase in intracellular acidity and accelerates the metabolic disorders of the cells (Trček et al. 2015; Geng et al. 2017). Great quantities of acetic acid may be released during biomass utilization in industrial production, which also leads to the increase in acid stress. Therefore, high acid tolerance capacity is indispensable for industrial strains, especially organic acid producers, and has become one of the most important standards for strain screening. Additionally, long-term use of probiotics is widespread in consumers with increasing awareness of nutritional requirements. During the process of food intake, stresses due to the abundance of gastric acids in the gastrointestinal tract are major survival challenges for probiotic microbes (Mills et al. 2011). In this context, several defense systems have been developed by microorganisms to survive the acid stress.

Sophisticated mechanisms at the physiological and molecular levels have been developed by microorganisms to survive and adapt to acid stress (Fernández-Niño et al. 2015; Hosseini Nezhad et al. 2015; Ju et al. 2016; Liu et al. 2015c; Matsui and Cvitkovitch 2010), and a variety of approaches has also been deployed to unveil acid tolerance mechanisms in different microbes at different levels (He et al. 2016; Hu et al. 2017; Lee et al. 2015; Sandoval et al. 2011; Zhai et al. 2014). After understanding the patterns and mechanisms of microbial response to acid stress comprehensively, specific strategies may be tailored for improvement of microbial producers and biosynthesis of valuable chemicals. Here, we systematically summarize recent progress in the study of microbial response to acidic stress and then discuss the industrial applications of the acid tolerance mechanisms. The introduction of systems and synthetic biology to identify acid resistance elements and engineer microbial cells for further enhanced acid resistance is outlined and prospected.

## Resistance mechanisms

### pH homeostasis

**Fig. 1:**
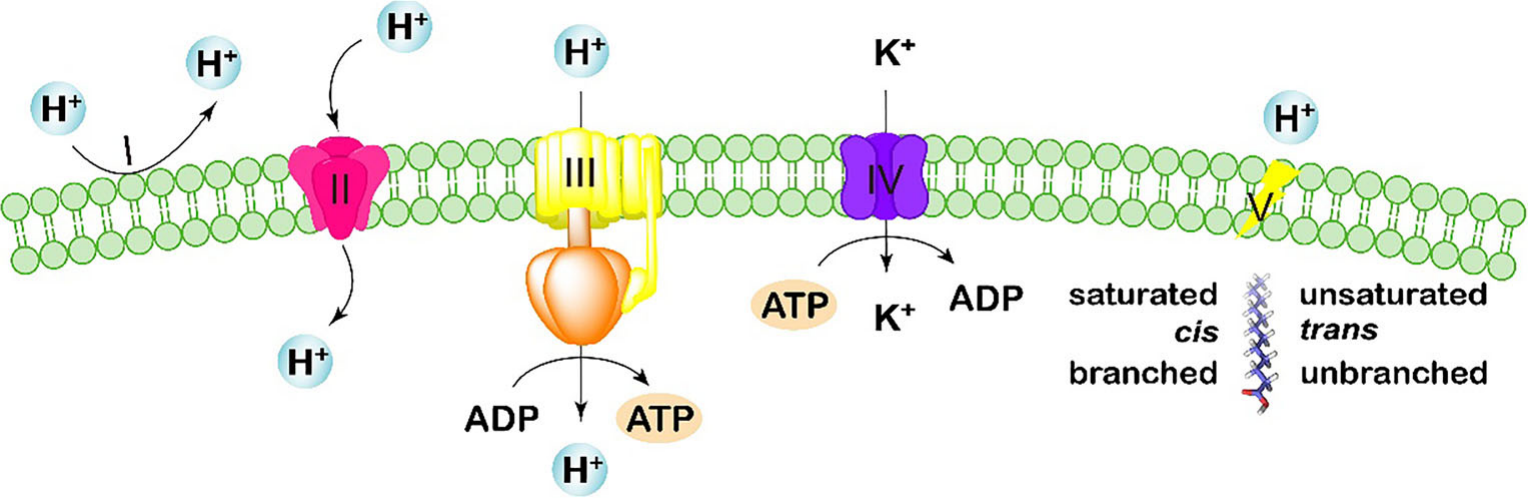
Acid tolerance mechanisms associated with cell membranes and ion transport systems. Microbial cells maintain pH homeostasis by restricting the inward flow of protons through highly impermeable cell membranes (I) and modulating the size of membrane channels (II), deflecting the influx of protons through generating chemiosmotic gradients via potassium ATPases (III), pumping excess protons out from the cytoplasm through proton pump (IV), and maintaining the integrity and fluidity of cell membranes by modulating fatty acid composition (V)

**Fig. 2:**
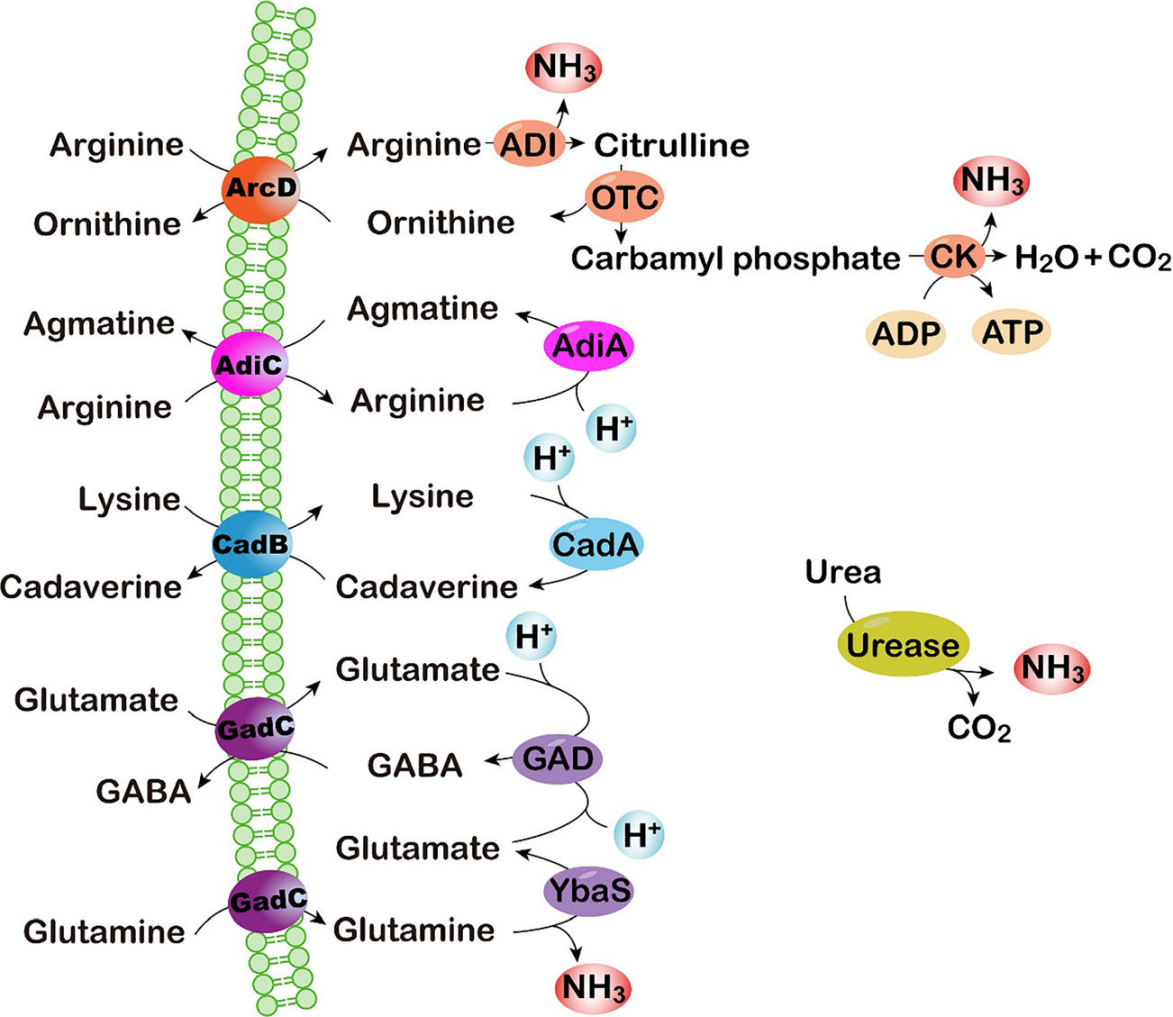
Enzyme-based acid tolerance mechanisms

pH homeostasis is the regulation of the pH inside and outside the cell and is an important indicator of the physiological state of cells in an acidic environment (Baker-Austin and Dopson 2007). It is critical for cell growth and metabolism, influencing the absorption and utilization of nutrients, the degradation of substrates, and the synthesis of proteins and nucleic acids (Guan et al. 2013). As illustrated in Figs. 1 and 2, the maintenance of pH homeostasis is a result of interactions among multiple transport systems. Electrogenic proton pumps expel protons from cells, generating a membrane potential and a pH gradient. The interconversion of these is regulated by cation and proton transfer via secondary transporters (Călinescu et al. 2014).

Different strategies to withstand acid stress by sustained pH homeostasis have evolved in microbes (He et al. 2017; Jain et al. 2013; Liu et al. 2016b; Lu et al. 2013; Miller and Maier 2014; Sohlenkamp 2017). Some yeast and bacteria maintain a relatively stable and neutral intracellular pH (pH_i_) in the presence of constantly changing extracellular pH (pH_ex_) and generate unfixed proton gradients (Siegumfeldt et al. 2000). However, a constant pH gradient is more favorable to most acid-tolerant microbes. This is because a large amount of energy must be consumed to maintain neutral pH_i_, which severely restricts the growth and metabolism of microbes (Sun 2016). The pH_i_ of these acid-tolerant microbes decreases with acidification of the environment, but is maintained at a higher level than pH_ex_. Once the acid reaches a certain concentration, the pH_i_ declines sharply, and the pH homeostasis is destroyed. This results in protein and DNA damage, with the cells ultimately withering (Wu et al. 2012a). Therefore, sustaining pH homeostasis is essential for microbes to survive in acidic environments.

#### Restriction of proton permeation

Proton motive force (PMF) is a measurement of the energy state of the cell membrane generated by a charge separation between the cytoplasm and external milieu created by membrane potential and pH gradient across the membrane (Baker-Austin and Dopson 2007). It is a common indicative reference for controlling pH homeostasis, which is mainly served by pH gradient in the study of acid resistance (Lee and Kang 2016). It is sustained by the balance between the inflow and outflow of protons.

Protons travel into the cytoplasm through the plasma membrane and are restricted by the proton permeability and channel size of the membrane (Sohlenkamp 2017). Acid-tolerant microbes are generally equipped with less permeable membranes to reduce the entry of protons into the cells (Sohlenkamp 2017). It is suggested that several factors contribute to this feature, including the tough structure of the monolayer, the bulky isoprenoid core, and a unique lipid composition such as tetraether lipids (Macalady and Banfield 2003). Modulating the size of membrane channels is another important strategy adopted by some acid-tolerant microbes to maintain pH homeostasis. Expression of the outer membrane porin of *Acidithiobacillus ferrooxidans* increased in response to acid, attempting to control the size of the porin gateway by forming a large L3 loop (Amaro et al. 1991). Consequently, the influx of protons was limited to only the outer membrane (Guiliani and Jerez 2000).

The influx of protons can also be reduced in acid-tolerant microbes using a chemiosmotic gradient generated by a Donnan potential, and the difference in electric potential formed between two solutions separated by an ion-exchange membrane without any current flow through the membrane (Baker-Austin and Dopson 2007). Many cation transporters were discovered in acidophiles, and they are presumed to be involved in the generation of a Donnan potential (Fütterer et al. 2004). Potassium transporters are reported to be the most efficient in generating chemiosmotic gradients, through which a reverse membrane potential is generated, and the inward flow of protons is restrained (Suzuki et al. 1999). It was also observed that potassium ions participate in the respiration-linked proton pump in *Sulfolobus* spp. (Schäfer 1996). In addition, cation ATPases (such as K^+^-ATPase) are involved in the maintenance of pH homeostasis by exchanging H^+^ and K^+^ (Macpherson et al. 2005).

An interesting acid resistance mechanism of some bacteria is the formation of biofilms. It is a group behavior which involves cell to cell communication (Li et al. 2001). Biofilms protect microbial cells against acid shock through wrapping the cells in the innermost part. Hence, cell density, which is related to the formation of biofilms, is also a factor affecting the acid resistance of microorganisms (Liu et al. 2015c).

#### Enhancement of proton pumps

The PMF-dependent proton pump is one of the most important acid tolerance systems in bacteria in the maintenance of pH homeostasis, through which excess protons are pumped out from the cytoplasm (Jain et al. 2013). Several proton pumps have been shown to promote proton efflux, such as the H^+^-ATPase, symporter, antiporter, and secondary transporter (Sun 2016). Protons are reported to be exported from cells through H^+^-ATPase in bacteria, a process that consumes ATP (Sun 2016). Consequently, higher H^+^-ATPase activity and more energy accumulation enhance the ability of cells to regulate pH_i_ homeostasis.

Normally, ATP is generated via F_o_F_1_ATPase when extracellular protons cross the cell membrane into the cytoplasm through a pH gradient (Sun 2016). However, the accumulation of H^+^ leads to a sharp decrease in pH_i_ under low pH_ex_, and proton pumps begin ATP consumption (Fig. 1). Consequently, the energy available for cells is depleted, and the survival of the strain is inhibited (Zheng et al. 2011). Therefore, elevating the energy levels is an effective strategy to enhance proton pumps. Substrate-level and oxidative phosphorylation are the two ways in which microorganisms produce ATP; the latter can be enhanced by adding auxiliary energy substrates (Zhou et al. 2009). It is reported that citrate is of significance in some prokaryotic microorganisms as an auxiliary energy cosubstrate, promoting ATP regeneration (Drici et al. 2010; Kang et al. 2013). Zhou et al. were able to increase ATP supply to *Candida glabrata* by adding citrate to the medium and increasing pH gradient of the system, thus improving its acid tolerance during pyruvic acid production (Zhou et al. 2011). In short, balancing proton transport and ATP metabolism forms the core of the proton pump mechanism. Besides for bacteria and yeast, *Rhizopus oryzae* has also been reported to resist acid stress through F_o_F_1_ATPase (Liu et al. 2015b).

#### Consumption of protons

In addition to controlling the transmembrane proton transport, some microorganisms have developed several acid tolerance mechanisms based on the consumption of excessive cytoplasmic protons to sustain pH homeostasis in acidic environments. The enzyme systems of cells that generate alkaline products play key roles in these mechanisms, as illustrated in Fig. 2.

The urease system is known to neutralize H^+^ by producing ammonia, which helps resist low pH during the culture of bacteria such as *Helicobacter pylori* (Mols and Abee 2011; Zanotti and Cendron 2010). Three models of urease have been proposed to regulate pH homeostasis. Originally, it was believed that urea is catalyzed by cell-associated extracellular urease and yields ammonia, which neutralizes protons around the cells (Hazell 1991). However, urease was later found to be a cytoplasmic enzyme that is released via cell lysis (Scott et al. 1998). According to the second model, ammonia produced from urease combines with H^+^ in the periplasm and the intracellular microenvironment is maintained by increasing pH of the same. The current generally accepted mechanism is that urease transforms urea into ammonia and CO_2_, directly neutralizing protons and regulating pH_i_ in the cytoplasm (Miller and Maier 2014). Vollan et al. found the role of *H. pylori* outer membrane phospholipase A in acid tolerance based on urea influx and ammonia efflux. This was later found to be involved in the transporting of NH_4_^+^ into periplasm (Vollan et al. 2017).

Amino acids render several microorganisms acid-tolerant by raising the pH_i_ during metabolism (Senouci-Rezkallah et al. 2011). Such systems have been termed amino acid-dependent acid tolerance systems. The arginine deaminase (ADI) system has been identified as an important defense mechanism in several bacteria against damage by acid (Liu et al. 2015c; Shabayek and Spellerberg 2017). Three steps are involved in this system (Fig. 2). First, arginine transported into cells by ArcD is converted to citrulline and ammonia by ADI. Next, ornithine carbamoyltransferase (OTC) catalyzes the phosphorolysis of citrulline to ornithine and carbamoyl phosphate. The former is subsequently transported out of the cell, while the latter is finally converted to carbon dioxide and ammonia by carbamate kinase (CK), during which ATP is generated from ADP. Consequently, protons are neutralized by ammonia and carbon dioxide formed by the system, and the ATP produced is available to extrude protons through H^+^-ATPase (Guan et al. 2013). Meanwhile, an arginine-agmatine antiporter AdiC and arginine decarboxylase AdiA comprise the other branch of the arginine-dependent acid tolerance system (Kanjee and Houry 2013). Arginine passes into the cell through AdiC and is converted to agmatine and carbon dioxide through catalysis by AdiA, consuming intracellular protons in the process.

The glutamate-dependent acid tolerance system is also recognized as critical for bacteria to survive in acidic environments. The function of the glutamate decarboxylase (GAD) system in acid resistance is similar to that of arginine decarboxylase (Fig. 2). Glutamate decarboxylase catalyzes the decarboxylation of glutamate, yielding γ-aminobutyric acid (GABA) and carbon dioxide, accompanied by proton consumption (Reeve and Reid 2016). The specific amino acid antiporter GadC, which is also known to transport glutamine, transports extracellular glutamate and intracellular GABA (Laroute et al. 2016; Ma et al. 2012). Another system, comprising GadC and the glutaminase YbaS, is found in *Escherichia coli* (Lu et al. 2013). After being transported into the cytoplasm, glutamine is converted to glutamate and ammonia by acid-activated YbaS, following which the GAD system is initiated. Formation of alkaline products (ammonia and GABA) and the reduction of intracellular protons are the net consequences of this glutamate-related metabolism. Besides arginine and glutamate, the lysine-dependent system also plays a role in acid tolerance of cells via the decarboxylation of lysine (He et al. 2017) (Fig. 2). In addition, some other amino acids such as aspartate and citrulline are involved in the maintenance of pH_i_ homeostasis by releasing ammonia during metabolism (Cusumano and Caparon 2015; Hu et al. 2010).

### Alteration of cell membranes

The primary target of environmental stress is cell membranes, which assist in sustaining cellular activities under acidic conditions in several ways. In addition to restricting proton permeation by adjusting channel size, membrane bioenergetics and lipid physiology are also closely related to the stress response in microorganisms (Yang et al. 2014). As mentioned above, the membrane-bound H^+^-ATPase regulates pH_i_ of cells by pumping protons out of the cytoplasm. Therefore, higher levels of H^+^-ATPase and its activity result in higher acid tolerance capacity (Zhang and Yang 2009). Modulation of the integrity, fluidity, and lipid composition of cell membranes are also important mechanisms that protect bacteria against the deleterious effects of acids (Yan et al. 2016).

Cell membranes provide a constant intracellular environment for cell growth and metabolism (Sohlenkamp 2017). Maintenance of proper membrane structure and function is a prerequisite for all cellular metabolic activities. Low pH usually leads to morphological changes in cells, which is a consequence of the damaged lipoidal cell membrane and decreased fluidity (Streit et al. 2008). The viability of cells under stress conditions is regulated by membrane status; cell membranes confer acid tolerance to cells through maintenance of their integrity and fluidity because of acid adaptation (Sohlenkamp 2017). Membrane fluidity is an integrated reflection of chain conformation, lateral and rotational diffusion, and resistance to sheer forces, and these characteristics are determined by the fatty acyl chain and head-group composition (Denich et al. 2003).

Some microbes regulate membrane fluidity by modulating fatty acid composition, since the bilayer structure can be modified by changing the distribution of fatty acids (Lindberg et al. 2013; Yang et al. 2014). The ratios of unsaturated to saturated, *cis* to *trans* unsaturated, and branched to unbranched fatty acids are all related to the acyl chain structure of glycerophospholipids. Altering the unsaturation ratio is a common mechanism employed by bacteria to control membrane fluidity. This depends on fatty acid synthesis by fatty acid synthases of the anaerobic pathways and desaturase enzymes of the aerobic pathways (Denich et al. 2003). It has been reported that higher unsaturation ratios of membrane fatty acids contribute to cell survival at low pH (Wu et al. 2012b). Isomerization of unsaturated fatty acids from *cis* to *trans* conformation also affects fluidity of the bacterial membrane (Tan et al. 2016). It is an energy-efficient post-synthesis lipid modification process, which occurs only in inactive cells (Diefenbach et al. 1992). Additionally, altering either the proportion or type of branching is another way in which cells modulate membrane fluidity (Kaiser et al. 2016; Sen et al. 2015). Specifically, membrane cyclopropane acyl chains were shown to be critical factors in acid tolerance in bacteria (Chang and Cronan 1999; Yang et al. 2015), where strains lacking such fatty acids were more sensitive to low pH (Kim et al. 2005). In addition, fatty acid chain length also plays a vital role in the response to acid stress. Strains reduce acid-mediated damage to their cell membranes by lengthening their fatty acid chains (Wu et al. 2012b).

### Metabolic regulations

Microorganisms have developed complex metabolic regulatory mechanisms to improve their acid tolerance during adaptation to acid environments. They upgrade their precursors, cofactors, and redox factors for survival, growth, and metabolism under acidic conditions by strengthening the glycolytic pathway (Guan et al. 2014). In a previous study, the glycolytic rate increased by 70% from pH 6.6 to 4.7 (Even et al. 2003), through changing enzyme concentrations and metabolic regulation of enzyme activities. The increase in enzyme activity compensates for the inhibition imposed by diminished pH, and rescues normal metabolism. Simultaneously, the transcription of central metabolic pathway genes is regulated and transcript stability increases. The increase in the enzyme pool and decrease in mRNA concentrations indicate that translational regulation plays a major role in enhancing enzyme concentrations by controlling ribosome activity (Even et al. 2003).

Glycolytic rates increased by 70%, and biomass synthesis was 80% less efficient at low pH, suggesting that the energy required in maintaining the metabolism of strains increased (Even et al. 2003). A portion of the energy that is consumed assists proton pumps in the maintenance of pH_i_ by extruding protons out of the cells. However, the available metabolic energy is limited since the rate of energy synthesis decreases upon cytoplasmic acidification. Thus, endogenous RNAs are catabolized to provide bases and ribose for the synthesis of carbon chains and energy (Siegumfeldt et al. 2000). Furthermore, amino acid catabolism is enhanced by fivefold when pH decreases from 6.6 to 4.7. The generation of NH_3_ and the consumption of intracellular H^+^ via deamination and decarboxylation, respectively, are considered key mechanisms in bacterial resistance to acidification (Lu et al. 2013; Xiong et al. 2014). Similarly, the metabolism and accumulation of cellular polyamines are also enhanced to promote cell survival in acidic pH (Fujihara and Yoneyama 1993).

Except for the protective mechanisms against protons, acid-resistant mechanisms based on anions from the dissociation of organic acids have also been developed. The consumption of acetate has been found to enhance acetic acid tolerance of *S. cerevisiae* (Geng et al. 2017). Through expression of genes in acetate degradation pathway, resistance of *S. cerevisiae* to acetic acid was improved during fermentation (Ding et al. 2015b). That is, anions may improve acid tolerance by involving in certain metabolic pathways and influencing the metabolism of acids.

### Protection and repair of macromolecules

An acid response mechanism that depends on protein synthesis has been widely observed in microorganisms (Liu et al. 2015c). Specific proteins are usually induced by acid stress to protect or repair macromolecules such as DNA and proteins. Several chaperones have been recognized as important acid tolerance factors, which are important during the synthesis, transport, folding, and degradation of proteins (Nicolaou et al. 2010).

In the periplasm of Gram-negative bacteria, the enzymes, transporters, and transmembrane antiporters encounter more severe acid stress because they lack the protection of the inner membrane. This leads to their denaturation and aggregation (Hong et al. 2012). HdeA and HdeB are two periplasmic chaperones that have been identified to protect enteric bacteria from damage by gastric acid, while HdeA also protects bacteria against acid stress due to accumulated organic acids (Mates et al. 2007). HdeA prevents the acid-induced aggregation of proteins by binding to them at an acidic pH, which is the condition in which the chaperone is activated (Tapley et al. 2009). HdeA is also involved in protein resolubilization and renaturation (Malki et al. 2008; Tapley et al. 2010). These proteins include transport proteins, metabolic enzymes, chaperones, lipoproteins, and proteases. Chaperones such as DegP and SurA can assist HdeA to protect proteins at low pH (Hong et al. 2012). They assist the recovery of protein activity by facilitating refolding during renaturation. HdeB is also an acid stress chaperone with the same functions as HdeA, although the optimum pH is different (Kern et al. 2007). HdeA and HdeB were recognized as the molecular chaperones that function specifically in acid tolerance (Hong et al. 2012).

Lo18 is a small membrane-associated heat shock protein that was characterized in *Oenococcus oeni* (Delmas et al. 2001). It improves the acid tolerance of bacteria through effectively suppressing protein aggregation, and it functions as a molecular chaperone to stabilize membrane and envelope proteins under acidic conditions (Weidmann et al. 2017). Ffh is a 54 kDa homolog of the signal recognition particle (SRP) complex, which is an essential component of the protein translocation pathway involved in membrane and extracellular protein transport (Gutierrez et al. 1999). It is part of the acid tolerance response system, and its transcription is regulated by pH. The lack of Ffh in *Streptococcus mutans* was found to lead to reduced H^+^-ATPase activity against a pH 5.0 shock (Kremer et al. 2001). In addition, several other chaperones such as DnaK, DnaJ, GrpE and HrcA, GroEL and GroES, Clp proteases, and EF-Tu have been shown to facilitate the repair of proteins as molecular chaperones during acid stress (Shabayek and Spellerberg 2017).

Depurination and depyrimidination of DNA can occur because of intracellular acidification, since protonation of a base can lead to cleavage of the glycosyl bond (Calhoun and Kwon 2011). DNA repair systems have been identified in microbial cells to survive DNA damage against low pH. *recA* encodes a multifunctional enzyme involved in synapsis, during which the paired DNA exchange strands (Adikesavan et al. 2011). The enzyme participates in DNA recombinational repair in *E. coli*, *Bacillus subtilis*, and *H. pylori*, along with RecN and AddAB (exonuclease V) (Ansari and Yamaoka 2017; Cardenas et al. 2014). The nucleotide excision repair system functions on damaged DNA produced from base modification, single-strand break, and abasic sites, and are considered the most important DNA repair system (Kisker et al. 2013). UvrABCD, DNA polymerase, and DNA ligase support the repair of acid-induced DNA damage, performing damage recognition, base excision, and gap filling (Das et al. 2015). UvrA overexpression enhanced the acetic acid tolerance and fermentation of *Acetobacter pasteurianus*, which is a widely used vinegar-brewing acetic acid bacteria (Zheng et al. 2018). In conclusion, the repair of damaged proteins and DNA is widely used by microbes to resist acid stress.

**Fig. 3:**
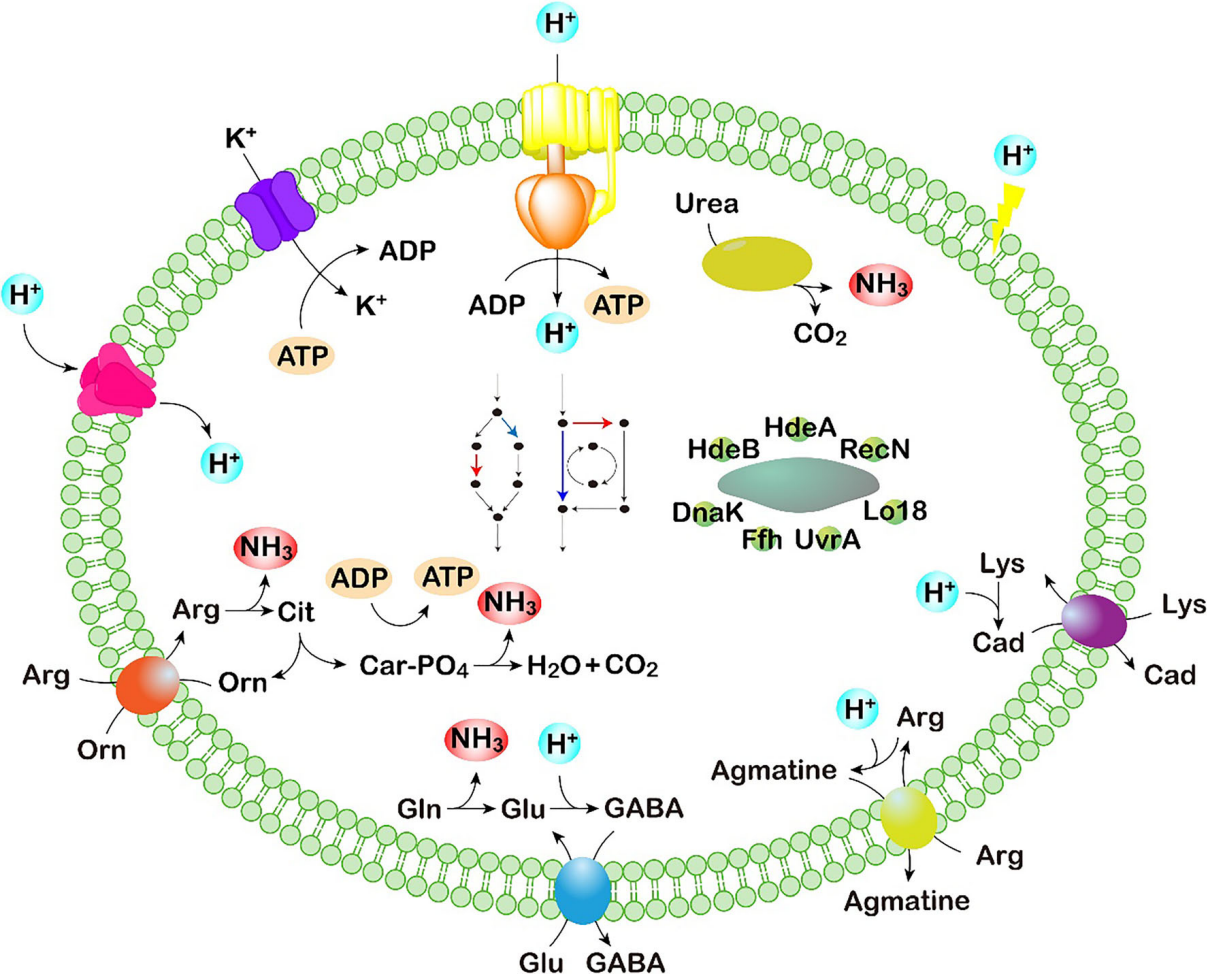
Acid stress responses in microbial cells

These mechanisms are mostly shared by various types of microorganisms. Additionally, the difference in cellular structure between prokaryotic and eukaryotic cells introduces diversity in acid-tolerant mechanisms. As eukaryote-specific organelles, mitochondria, vacuole and nucleus all play roles in acid tolerance of *S. cerevisiae* (Peng et al. 2017). Acid-tolerant mechanisms utilized by different microorganisms were summarized in Fig. 3 and listed in Table 1, respectively.

**Table 1:** Acid-tolerant mechanisms utilized by various microorganisms

Mechanisms	Bacteria	Yeasts
F_0_F_1_-ATPase proton pumps	*E. coli* (Foster 2004; Sun et al. 2012b) *Lactococcus* (O’Sullivan and Condon 1999) *Lactobacillus* (Koponen et al. 2012) *Streptococcus* (Martin-Galiano et al. 2005; Kuhnert and Quivey 2003) *Corynebacterium glutamicum* (Jakob et al. 2007) *P. acidipropionici* (Zhang and Yang 2009; Guan et al. 2013) *Bacillus* (Shobharani and Halami 2014)	*S. cerevisiae* (Casal et al. 2016) *C. glabrata* (Zhou et al. 2011) *Zygosaccharomyces bailii* (Palma et al. 2015)
Decarboxylation and deamination	*E. coli* (Iyer et al. 2003; Sun et al. 2012a; Lu et al. 2013) *Lactococcus* (Budin-Verneuil et al. 2006) *Lactobacillus* (Su et al. 2011) *P. acidipropionici* (Guan et al. 2013)	
Cell membrane modification	*E. coli* (Chang and Cronan 1999) *Lactococcus* (Wu et al. 2012b) *Lactobacillus* (Broadbent et al. 2010)	*S. cerevisiae* (Ding et al. 2009; Zhao and Bai 2009) *Z. bailii* (Palma et al. 2015)
Metabolic regulation	*P. acidipropionici* (Guan et al. 2014)	*S. cerevisiae* (Wu et al. 2016) *Z. bailii* (Palma et al. 2015)
Macromolecule protection and repair	*E. coli* (Hong 2012; Mujacic and Baneyx 2007) *Lactococcus* (Weidmann et al. 2017) *Lactobacillus* (Koponen et al. 2012) *Streptococcus* (Shabayek and Spellerberg 2017) *C. glutamicum* (Jakob et al. 2007) *A. pasteurianus* (Zheng et al. 2018)	*S. cerevisiae* (Ding et al. 2009) *Z. bailii* (Palma et al. 2015)
Protection from organelle		*S. cerevisiae* (Ding et al. 2015a; Kumar et al. 2015; Cheng et al. 2016)

## Industrial applications of acid tolerance in microorganisms

### Enhanced survival of probiotics in the gastrointestinal tract

With improvement in quality of life, consumers are paying increased attention to their health. They now demand nutrition rather than just being adequately fed. Functional foods with potential health benefits are attracting increasing interest, wherein food-preserving microorganisms, especially probiotics, play significant roles. They do not only provide high levels of nutraceuticals to the food, but also participate in health regulation of humans by generation of functional molecules in situ in the gastrointestinal tract (Liu et al. 2017).

Several lactic acid bacteria and dairy propionibacteria have been generally regarded as safe, and fermented foods and oral agents containing lactic acid bacteria are developing rapidly as probiotics. They produce a number of valuable compounds including bacteriocins, exopolysaccharides, vitamins, and conjugated linoleic acids (Li and Cao 2010) and have potential health benefits including the regulation of intestinal motility and absorption, balance of intestinal microecology, reduction of inflammation, and the modulation of the immune system (Cousin et al. 2011).

The multiple health benefits of probiotics require that their metabolic activities and physiological functions are maintained in humans. The stress caused by gastric acid is one of the key challenges to their survival (Ranadheera et al. 2014). Extensive studies have revealed the acid tolerance mechanisms in these bacteria (Bustos et al. 2015; Guan et al. 2013; Shobharani and Halami 2014; Wu et al. 2012a). Through comparing acid tolerance characteristics such as H^+^-ATPase activity and cellular fatty acid profile, the acid tolerance of *Bacillus* sp. was assessed, which provided choices and a reference for industries and consumers as possible probiotics (Shobharani and Halami 2014). Chocolate processing has been used as an effective method to improve the acid tolerance of probiotics (*Labre*) and make them deliverable to the intestine (Yonejima et al. 2015). Milk was employed as a suspension medium to protect probiotics such as *Butyricicoccus pullicaecorum* from low pH during the initial phase of intake (Geirnaert et al. 2014). Based on the understanding of their response to acids, strategies can be developed to protect the probiotics against acid damage, and thus enhance their survival and physiological function in humans.

### Enhancement of organic acid production by acid-tolerant strains

Organic acids are important building block chemicals with increasing market demand. A variety of industrial applications has been developed for organic acids. Propionic acid (PA) is widely used in the organic synthesis of cellulose fiber, perfume, paint, herbicides, and pharmaceuticals (Liu et al. 2012). Propionibacteria are most commonly used for the biosynthesis of PA because of their vitality, high yields, capability to use a wide variety of substrates, and antimicrobial properties (Guan et al. 2015b). Various strategies have been developed to improve PA yield and productivity in propionibacteria, including the optimization of carbon sources and fermentation modes, controlling culture conditions such as pH, oxidoreduction potential, the reduction of byproduct accumulation, and the engineering of metabolic pathways (Feng et al. 2010; Liu et al. 2015a, 2016a; Wang et al. 2015; Zhuge et al. 2013, 2014, 2015). However, it cannot meet the industry requirements. It has been reported that the accumulation of PA strongly inhibits cell growth and metabolic activity during its fermentation by propionibacteria (Guan et al. 2016). Extractive fermentation and cell immobilization have been used to enhance PA production significantly (Zhu et al. 2012). However, high osmotic pressure and potential toxicity may be induced upon extractant addition, where the low productivity and high cost of cell-immobilized fermentation is also undesirable (Liu et al. 2012). Therefore, enhancing the acid tolerance of propionibacteria is considered an effective strategy for enhanced PA production.

Currently, evolutionary engineering approaches have been applied to improve the acid tolerance and PA production of propionibacteria through random mutation. Adaptive evolution is a powerful tool for strain improvement, during which the tolerant strains are repeatedly transferred into fresh broth and the pH is lowered gradually. The evolved strains showed higher yields and productivity of PA (Suwannakham and Yang 2005; Zhu et al. 2010). Another efficient technology of evolutionary engineering for rapid phenotype improvement is genome shuffling. Multiple superiority genes obtained from classical mutagenesis were recombined through recursive protoplast fusion. A mutant library of *P. acidipropionici* was constructed using ultraviolet irradiation and diethyl sulfate mutagenesis, followed by recursive protoplast fusion to allow recombination of genomes (Guan et al. 2012). After multiple rounds of protoplast fusion, an acid-tolerant strain was obtained, and the PA titer and productivity were enhanced by 33.3% and 65%, respectively. When compared to adaptive evolution and other classical strain improvement strategies, phenotypic improvement is faster and more efficient (Guan et al. 2012).

In the absence of known specific molecular mechanisms, the improvement of acid tolerance through evolutionary engineering is limited. With the development of genetic manipulation tools, reverse engineering of tolerant phenotypes provides opportunities for further improvement of acid tolerance. First, the key factors responsible for acid tolerance must be identified. In recent years, the acid tolerance mechanisms of propionibacteria have been investigated at different levels using omics techniques. It was revealed at the microenvironment level that *P. acidipropionici* maintains pH homeostasis under acid stress by enhancing H^+^-ATPase activity and intracellular energy status (Zhang and Yang 2009). The ADI and GAD systems were also found to aid the consumption of protons (Guan et al. 2013). The key proteins and metabolites involved in acid tolerance have been identified through comparative proteomic and metabolomic analyses of the wild type *P. acidipropionici* and its acid-tolerant mutants (Guan et al. 2014, 2015a). In addition, genomics and transcriptomics can unveil acid tolerance-related genes and transcriptional regulators. On these bases, metabolic engineering has been performed on *P. jensenii* to improve acid resistance and PA production through overexpressing the acid-resistant elements detected by system biology (Guan et al. 2016). In conclusion, the acid-tolerant mechanisms of propionibacteria have yet to be fully elucidated systematically. Improvements in acid tolerance can yet be made to enhance PA production through rational synthetic biology approaches, and engineering microbial cells at the genetic level.

Similar to PA synthesis by propionibacteria, production of other organic acids can also be enhanced by improving the acid tolerance of strains. It has been demonstrated that acid-tolerant strains are more effective in lactic acid production (Patel et al. 2006). Adaptive evolution and genome shuffling were also used to improve the acid tolerance of *Lactobacillus* (Patnaik et al. 2002; Zhang et al. 2012), whereby the production of lactic acid increased significantly. RNA-Seq transcriptomic analysis was performed to investigate the acid-resistant mechanisms of *Acetobacter pasteurianus*, providing more basics and opportunities for higher acid tolerance and acetic acid production (Yang et al. 2019). Similarly, omics were also introduced to analyze acetic acid tolerance in *Saccharomyces cerevisiae* (Geng et al. 2017). Improved acetic acid tolerance was obtained by modifying key genes identified, which is of great potential in industrial processes.

Acid stress is unavoidable for microorganisms during fermentation in the synthesis of the other products. Screening lactic acid bacteria with high GABA production has been performed. It is shown that low pH favors the activity of glutamate decarboxylase, which is important for GABA biosynthesis in lactic acid bacteria (Komatsuzaki et al. 2008). Thus, the acid-tolerant strains are most likely to produce high levels of GABA. Many studies based on the engineering of acid stress resistance in lactic acid bacteria have been performed to enhance the acid tolerance as well as lactic acid production. The glutathione synthetase genes from *E. coli* and the trehalose biosynthetic pathway from *P. freudenreichii* were expressed in *Lactococcus lactis* respectively to increase survival under acid stress (Carvalho et al. 2011; Zhang et al. 2007). The histidine decarboxylation pathway, which enables cells to survive at low pH, was also expressed in *L. lactis* (Trip et al. 2012). The betaine uptake system from *Listeria monocytogenes* was introduced into *Bifidobacterium breve* to increase resistance to gastric acid (Sheehan et al. 2007). The acid tolerance of *L. lactis* was enhanced by overexpressing molecular chaperone proteins DnaK (Tian et al. 2012) as well as the DNA repair protein RecO (Wu et al. 2013). Table 2 lists the genes involving in acid tolerance of microbes which have been verified through genetic manipulation.

**Table 2:** Genes engineered by different microorganisms for improving acid tolerance

Mechanisms	Genes	Microorganisms	Acid stress	References
F_0_F_1_-ATPase proton pumps	*AtMtATP6*	*S. cerevisiae*	Pyruvic acid	(Zhang et al. 2008)
	*atpA*	*P. acidipropionici*	Propionic acid	(Guan et al. 2014)
	*CgAMD1*	*C. glabrata*	Hydrochloric acid	(Wu et al. 2018)
Decarboxylation and deamination	*cad*	*E. coli*	Acetic acid	(Noh et al. 2018)
	*ybaS*, *gadC*	*E. coli*	Hydrochloric acid	(Lu et al. 2013)
	*hdcAPB*	*L. lactis*	Hydrochloric acid	(Trip et al. 2012)
	*arcA*, *arcC*, *gdh*, *gadB*, *ybaS*	*P. acidipropionici*	Propionic acid	(Guan et al. 2016)
Cell membrane modification	*cfa*	*E. coli*	Hydrochloric acid	(Chang and Cronan 1999)
	*murG*	*L. lactis*	Lactic acid	(Zhang et al. 2016)
	*FPS1*	*S. cerevisiae*	Acetic acid	(Zhang et al. 2011)
	*Yro2*, *Mrh1*	*S. cerevisiae*	Acetic acid	(Takabatake et al. 2015)
Metabolic regulation	*gshA*, *gshB*	*L. lactis*	Lactic acid	(Zhang et al. 2007)
	*trePP*, *pgmB*, *otsB*	*L. lactis*	Lactic acid	(Carvalho et al. 2011)
	*BetL*	*Bifidobacterium breve*	Gastric acid	(Sheehan et al. 2007)
	ACS2	*S. cerevisiae*	Acetic acid	(Ding et al. 2015b).
Macromolecule protection and repair	*dnaK*	*L. lactis*	Lactic acid	(Abdullah-Al-Mahin et al. 2010)
	*shsp*	*L. lactis*	Lactic acid	(Tian et al. 2012)
	*RecO*	*L. lactis*	Lactic acid	(Wu et al. 2013)
	*UvrA*	*A. pasteurianus*	Acetic acid	(Zheng et al. 2018)
Protection from organelle	*COX20*	*S. cerevisiae*	Acetic acid	(Kumar et al. 2015)
	*PEP3*	*S. cerevisiae*	Acetic acid	(Ding et al. 2015a)
	*RTT109*	*S. cerevisiae*	Acetic acid	(Cheng et al. 2016)

## Conclusions and prospects

**Fig. 4:**
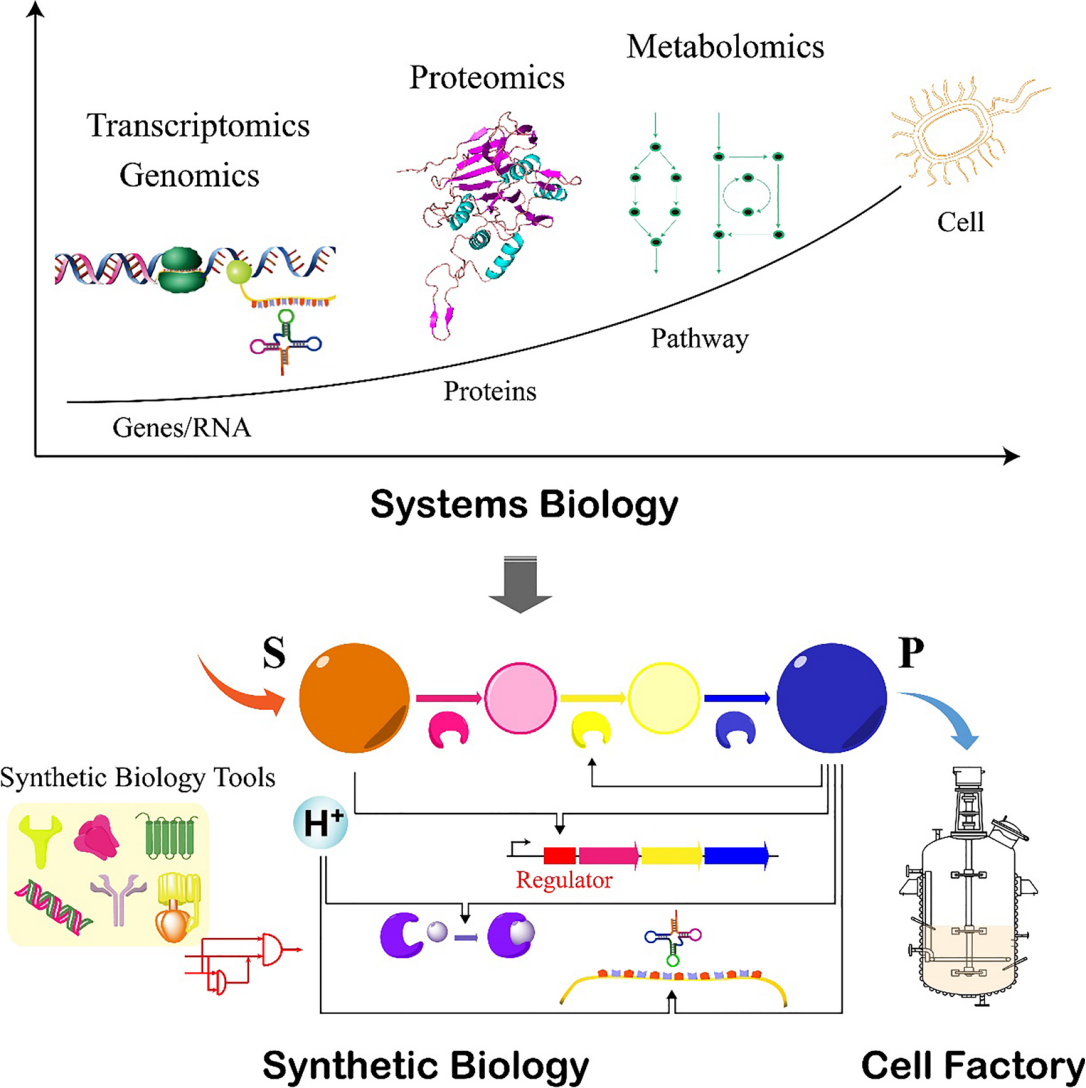
Introduction of systems and synthetic biology to construct microbial cell factories for the improved production of organic acids

Microbial cells suffer acid stress when ingested as probiotics or in the production of organic acids. Physiological and genetic resistance mechanisms have evolved in microorganisms to survive in acidic environments, including pH homeostasis, alteration of cell membranes, regulation of metabolism, and repair of macromolecules (Fig. 3). Although they share similar resistance mechanisms, different species utilize a variety of specific elements as a response to acid stress. Therefore, customized strategies must be proposed for different strains. Currently, the acid tolerance mechanisms of microbial cells have been used in industry for improved probiotic intake and organic acid production. To further enhance the performance of industrial microorganisms, the development of effective tools to improve tolerance mechanisms is critical. The combination of systems and synthetic biology provides significant opportunities to further enhance the acid tolerance of probiotics, and construct microbial cell factories for valuable chemicals (Fig. 4). A more comprehensive understanding of microbial acid tolerance mechanisms can be obtained using systems biology technologies, and thus specific acid-tolerant elements would be uncovered. These elements can then be targeted by synthetic biology tools for improved acid tolerance and mass chemical production.
